# A new surgical method of treatment spontaneous intracranial hemorrhage

**DOI:** 10.1515/tnsci-2020-0164

**Published:** 2021-04-20

**Authors:** Ning Du, Xinjun Wang, Xuyang Zhang, Jingwei Xie, Shaolong Zhou, Yuehui Wu, Yongkun Guo

**Affiliations:** Department of Neurosurgery, The Fifth Affiliated Hospital of Zhengzhou University, 3 Kangfuqian Street, Erqi District, Zhengzhou, Henan, People’s Republic of China

**Keywords:** intracranial hemorrhage, neuroendoscopy, diffusion-tensor imaging

## Abstract

**Objective:**

This study aimed to determine the safety and effectiveness of DTI-assisted neuroendoscopy for treating intracranial hemorrhage (ICH).

**Methods:**

This retrospective study included clinical data from 260 patients with spontaneous supratentorial ICH who received neuroendoscopic hematoma removal. Patients were separated into groups based on the surgery method they received: DTI-assisted neuroendoscopy (69 cases) and standard neuroendoscopy (191 cases). All patients were followed up for 6 months. Multivariate logistic regression analyzed the risk factors affecting the prognosis of patients. The outcomes of the two groups were compared using Kaplan–Meier survival curves.

**Results:**

The prognostic modified Rankin Scale (mRS) score was significantly better (*P* = 0.027) in the DTI-assisted neuroendoscopy group than in the standard neuroendoscopy group. Logistic regression analysis showed that DTI-assisted neuroendoscopy is an independent protective factor for a favorable outcome (model 1: odds ratio [OR] = 0.42, *P* = 0.015; model 2: OR = 0.40, *P* = 0.013). Kaplan–Meier survival curves were used to show that the median time for a favorable outcome was 66 days (95% confidence interval [CI] = 48.50–83.50 days) in the DTI-assisted neuroendoscopy group and 104 days (95% CI = 75.55–132.45 days) in the standard neuroendoscopy group. Log-rank testing showed that the DTI-assisted neuroendoscopy group had a lower pulmonary infection rate (*χ* ^2^ = 4.706, *P* = 0.030) and a better prognosis (*χ* ^2^ = 5.223, *P* = 0.022) than the standard neuroendoscopy group. The survival rate did not differ significantly between the DTI-assisted neuroendoscopy group and the standard neuroendoscopy group (*P* > 0.05).

**Conclusions:**

The use of DTI in neuroendoscopic hematoma removal can significantly improve neurological function outcomes in patients, but it does not significantly affect the mortality of patients.

## 1 Introduction

ICH (intracranial hemorrhage) accounts for 10–20% of strokes in Western countries and 18.8–47.6% of strokes in China [1]. Hemorrhagic strokes produce higher disability and mortality rates than do ischemic strokes. Studies have shown that the 30-day mortality rate in patients with a hematoma volume of more than 60 mL is as high as 91% [2].

The treatment methods for ICH remain controversial. The Surgical Treatment for Intracerebral Hemorrhage (STICH) I and II trials [3,4] found that routine craniotomy did not improve the neurological function outcomes or reduce mortality. Treatments for ICH have improved in recent years with the development of minimally invasive techniques such as neuroendoscopy and stereotactic surgery [5,6,7]. Taking into account the varying severity of cases in the STICH studies, the Minimally Invasive Surgery plus Alteplase in the Intracerebral Haemorrhage Evacuation (MISTIE) II and III studies have shown that minimally invasive surgery is safe and feasible for treating ICH. The mortality rate of patients has decreased, but their neurological function outcomes have not been significantly improved through this method [8,9]. Therefore, it is still worth investigating how the prognosis of patients with ICH can be improved, and how to reduce the social burden of this condition.

DTI (diffusion-tensor imaging) technology can specifically show the spatial relationships between white-matter fiber tracts and hematomas. Hsieh et al. [10] confirmed the value of using DTI in the treatment of ICH by using it to investigate the relationship between changes in the pyramidal tract before and after an ICH and the recovery of motor function. Therefore, using DTI technology during the treatment of ICH may be helpful to improve the prognosis of patients.

Previous clinical randomized controlled trials (RCTs) did not involve DTI technology. The aim of the present study was to determine the significance of DTI-assisted neuroendoscopy in the treatment of ICH, with the expectation of further reducing the disability rate and improving the prognosis of patients with ICH.

## 2 Materials and methods

### 2.1 Patient selection

The clinical data of 392 patients with ICH from January 2015 to December 2019 were analyzed retrospectively. This study only included patients with spontaneous supratentorial ICH aged 18–80 years, a hemorrhage volume of 20–50 mL, no brain herniation, and a GCS (Glasgow Coma Scale) score of >8. We did not consider secondary ICH caused by trauma or tumor, patients with pregnancy or other serious underlying diseases, or those with incomplete follow-up data. Among the 392 patients, 73 had a hemorrhage volume of >50 mL, 26 cases had a subtentorial ICH, 15 were older than 80 years, 8 had other serious conditions (2 with respiratory failure, 5 with circulatory failure, and 1 with kidney failure), 1 had been pregnant for 5 months, and 9 were not followed up or their families refused to provide clinical data. These 132 patients were excluded from the present study.


**Informed consent:** Informed consent has been obtained from family members of all individuals included in this study.
**Ethical approval:** The conducted research is not related to either human or animals use.

### 2.2 Data collection and analysis

The following data were collected and analyzed: age, sex, past medical history, history of smoking and alcohol abuse, laboratory parameters on admission, GCS score, bleeding site, hematoma volume, whether the hematoma broke into the ventricle, length of hospital stay, postoperative rebleeding, postoperative hydrocephalus, postoperative intracranial infection, postoperative pulmonary infection, postoperative urinary tract infection, and the prognosis at 6 months after surgery.

Based on the CT (computer tomography) scan obtained at admission, the volume of the ICH in milliliters was estimated based on its approximate elliptical volume using the ABC/2 formula, or the ABC/3 formula for irregularly shaped ICHs [11,12,13]. Rebleeding was defined as a 30% increase in the volume of hematoma or bleeding >6 mL [14]. Intracranial infection, hydrocephalus, pulmonary infection, and urinary tract infection were judged according to established standards [15,16,17,18].

### 2.3 Treatment methods

**Figure 1:**
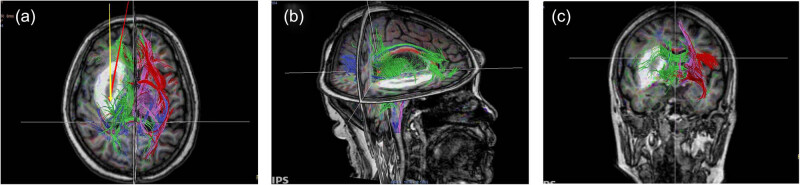
DTI (diffusion-tensor imaging) shows the spatial relationships between white-matter fiber tracts and hematomas. In the picture (a), the red arrow represents the conventional surgical channel (the long axis of the hematoma); the yellow arrow represents the new surgical channel with the assistance of DTI and guidance of the stereotactic navigation system. The picture (b) and (c) show the spatial relationships between the hematoma and the white-matter fiber tracts at different angles.

The patients in the DTI-assisted neuroendoscopy group were given portable electrocardiography (ECG) monitors and oxygen packs and were accompanied by clinicians when a rapid DTI examination was performed before the operation. We upload the image data to the stereotactic neuronavigation system. Using basal ganglia hematoma as an example, the formation of a hematoma in the basal ganglia area could be seen in the obtained images, some nerve fiber tracts were interrupted, and the remaining nerve fiber tracts were in a semienclosed relationship with the hematoma. In order to avoid iatrogenic damage to nerve fiber tracts and remove the hematoma as successfully as possible, we selected the largest slice of the hematoma and, guided by the stereotactic navigation system, tilted the conventional surgical channel (the long axis of the hematoma) to the outside to avoid as much bundling of the nerve fibers in the frontal lobe as possible (shown in Figure 1).

The patient was placed in a supine position and received endotracheal intubation, general anesthesia, and routine disinfection drapes around the surgical incision. A straight incision of 4–5 cm was made along a marked surgical line, a scalp retractor was fixed, and the skull was drilled while maintaining hemostasis using bone wax, with the dura cut in a cross shape. Puncturing by a disposable brain cannula was performed under guidance from a stereotactic neuronavigation system. After the cannula reached the hematoma cavity, the tube core was pulled out, the cannula was inserted, and the intracranial hematoma was carefully removed while viewing via a neuroendoscope. After the hematoma was removed and hemostasis was achieved through electrocoagulation, the surgical field was covered with a gelatin sponge. It was then necessary to insert and fix a drainage tube, withdraw the cannula, suture the dura mater, fix the bone flap using the connecting piece, and suture the skin flap. The operation ended after all these steps had been completed, and the drainage tube was removed at a time based on the amount of residual hematoma.

A conventional surgical approach was adopted for patients in the standard neuroendoscopy group which was the same as that used in the DTI-assisted neuroendoscopic procedure.

All surgery patients were routinely monitored postoperatively using ECG to maintain a stable blood pressure and other vital signs, to reduce dehydration and the intracranial pressure, prevent infection, nourish nerves, and supplement water, electrolytes, and nutrition. A review CT scan was obtained in all patients within 24 h of surgery. Early rehabilitation therapy commenced after the patient had reached a stable condition.

### 2.4 Outcome measures

All patients were followed up for 6 months through outpatient follow-ups, inpatient reviews, and telephone follow-ups. mRS (modified Rankin Scale) scores of 0–3 and 4–6 were defined as favorable and unfavorable outcomes, respectively [19].

### 2.5 Statistical analysis

All statistical analyses were performed using standard statistical software (SPSS, version 24.0). The measurement data were first tested for normality using the Kolmogorov–Smirnov test, and they are presented as mean ± SD values (which were compared using Student’s *t* test) or median and inter-quartile-range values (which were compared using the Mann–Whitney *U* test), as appropriate. Pearson’s *χ* ^2^ test or Fisher’s exact test was applied to categorical variables, which were expressed as number (percentage) values. All single factors for which *P* < 0.1 were included in the multivariate logistic regression model when analyzing the effect of treatment on the prognosis. Further analyses of the influence of surgical methods on the prognosis were performed by producing Kaplan–Meier survival curves. The significance cutoff was set at *α* = 0.05, and all statistical tests were two-sided.

## 3 Results

### 3.1 Comparison of the effects of the two surgical interventions on intracranial hemorrhage

There were no significant differences between the two surgical interventions in sex, age, past medical history (history of smoking and alcohol abuse, stroke, hypertension, diabetes, hyperlipidemia, antihypertensive medication, and anticoagulant medication), laboratory parameters, hematoma volume, whether the hematoma broke into the ventricle, length of hospital stay, or the GCS score at admission (all *P* > 0.05). However, patients in the DTI-assisted neuroendoscopy group had a significantly higher blood glucose level (*P* < 0.05) (shown in Table 1).

**Table 1:** Comparison of clinical data between DTI-assisted neuroendoscopy group and neuroendoscopy group

Variable	DTI-assisted neuroendoscopy group (*n* = 69)	Neuroendoscopy group (*n* = 191)	*P*
Age (X ± S, year)	60.2 ± 12.7	60.0 ± 11.7	0.905
Male, *n* (%)	46 (66.7)	116 (60.7)	0.383
Smoking, *n* (%)	27 (39.1)	66 (34.6)	0.497
Alcohol abuse, *n* (%)	22 (31.9)	59 (30.9)	0.879
**Past medical history**			
History of stroke, *n* (%)	15 (21.7)	33 (17.3)	0.413
Hypertension, *n* (%)	50 (72.3)	130 (68.1)	0.497
Diabetes, *n* (%)	9 (13.0)	20 (10.5)	0.561
Coronary heart disease, *n* (%)	10 (14.5)	24 (12.6)	0.684
Hyperlipidemia, *n* (%)	14 (20.3)	28 (14.7)	0.276
Hypotensor, *n* (%)	42 (60.9)	114 (59.7)	0.863
Warfarin treatment, *n* (%)	13 (18.8)	26 (13.6)	0.297
**Laboratory examination**			
Lymphocyte, median (IQR), 10^9^/L	0.92 (0.59–1.25)	0.97 (0.67–1.46)	0.239
Leucocytes, median (IQR), 10^9^/L	9.67 (7.90–13.01)	10.48 (7.74–13.07)	0.458
Blood glucose, median (IQR), 10^9^/L	9.26 (7.75–11.13)	8.02 (6.90–9.60)	0.007
Albumin, median (IQR), 10^9^/L	38 (35–44)	40 (36–44)	0.113
Hemoglobin, (X ± S, 10^9^/L)	134.1 ± 19.2	130.1 ± 18.9	0.135
INR, median (IQR), 10^9^/L	0.92 (0.85–1.05)	0.94 (0.86–1.02)	0.513
Hematoma volume, median (IQR), mL	39 (26–46)	34 (26–40)	0.053
**ICH location**			
Basal ganglia, *n* (%)	61 (88.4)	144 (75.4)	0.023
Lobar, *n* (%)	8 (11.6)	47 (24.6)	0.023
Broke into the ventricle, *n* (%)	15 (21.7)	51 (26.7)	0.417
GCS score, median (IQR)	9 (8–9)	9 (8–10)	0.191
**Prognosis**			
mRS score, median (IQR)	3 (2–4)	3 (2–5)	0.027
Rebleeding, *n* (%)	12 (17.4)	25 (13.1)	0.381
Hydrocephalus, *n* (%)	5 (7.2)	17 (8.9)	0.672
Intracranial infection, *n* (%)	6 (8.7)	15 (7.9)	0.826
Pulmonary infection, *n* (%)	16 (23.2)	70 (36.6)	0.042
Urinary tract infection, *n* (%)	6 (8.7)	8 (4.2)	0.210^a^
Hospital stay, median (IQR), day	17 (12–27)	19 (13–31)	0.874
Mortality, *n* (%)	11 (15.9)	46 (24.1)	0.161

IQR: inter-quartile range; ICH: intracerebral hemorrhage; GCS: Glasgow coma scale; INR: international normalized ratio; DTI: diffusion tensor imaging.

a Fisher’s exact test.

The pulmonary infection rate was lower and the prognosis was significantly better in the DTI-assisted neuroendoscopy group than in the standard neuroendoscopy group. In the DTI-assisted neuroendoscopy group, 49 cases (71.0%) had a favorable outcome in terms of prognostic mRS scores and there were 16 cases (23.2%) of lung infection; the corresponding values in the standard neuroendoscopy group were 109 cases (57.1%, *P* = 0.027) and 70 cases (36.6%, *P* = 0.042), respectively. There were no significant differences in mortality or postoperative complications (rebleeding, hydrocephalus, intracranial infection, and urinary tract infection) between the two groups (all *P* > 0.05) (shown in Table 1).

### 3.2 Comparative analysis of factors affecting the prognosis of patients with intracranial hemorrhage

After dividing the study patients into a favorable-outcome group and an unfavorable-outcome group, there were no significant between-group differences in sex, age, past medical history (history of smoking and alcohol abuse, diabetes, history of hyperlipidemia, history of antihypertensive drugs, and history of anticoagulants), laboratory parameters, or bleeding site (all *P* > 0.05). The factors associated with a good prognosis in the ICH patients were no history of stroke, history of hypertension, hematoma breaking into the ventricle, postoperative rebleeding, or pulmonary infection, a small hematoma volume, a high GCS score, and preoperative DTI assistance (all *P* < 0.05) (shown in Table 2).

**Table 2:** Comparison of clinical data between favorable and unfavorable outcome groups

Variable	Favorable outcome mRS = 0–3 (*n* = 158)	Unfavorable outcome mRS = 4–6 (*n* = 102)	*P*
Age, median (IQR), year	58.5 (52.0–69.0)	62.0 (51.8–70.0)	0.675
Male, *n* (%)	103 (65.2)	59 (57.8)	0.233
Smoking, *n* (%)	62 (39.2)	31 (30.4)	0.146
Alcohol abuse, *n* (%)	55 (34.8)	26 (25.5)	0.113
**Past medical history**			
History of stroke, *n* (%)	22 (13.9)	26 (25.5)	0.019
Hypertension, *n* (%)	117 (74.1)	63 (61.8)	0.036
Diabetes, *n* (%)	21 (13.3)	8 (7.8)	0.173
Coronary heart disease, *n* (%)	24 (15.2)	10 (9.8)	0.208
Hyperlipidemia, *n* (%)	27 (17.1)	15 (14.7)	0.610
Hypotensor, *n* (%)	100 (63.3)	56 (54.9)	0.178
Warfarin treatment, *n* (%)	28 (17.7)	11 (10.8)	0.126
**Laboratory examination**			
Lymphocyte, median (IQR), 10^9^/L	0.93 (0.65–1.35)	0.94 (0.68–1.53)	0.403
Leucocytes, median (IQR), 10^9^/L	10.02 (7.90–12.93)	10.71 (7.72–13.69)	0.307
Blood glucose, median (IQR), 10^9^/L	8.12 (7.10–9.60)	8.69 (7.23–10.65)	0.366
Albumin, median (IQR), 10^9^/L	38 (34–44)	40 (37–44)	0.103
Hemoglobin, median (IQR), 10^9^/L	133.5 (118.8–147.0)	132.0 (115.9–144.1)	0.284
INR, median (IQR), 10^9^/L	0.93 (0.86–1.04)	0.93 (0.87–1.02)	0.631
Hematoma volume, median (IQR), mL	32 (25–40)	40 (30–45)	0.000
**ICH location**			
Basal ganglia, *n* (%)	125 (79.1)	80 (78.4)	0.895
Lobar, *n* (%)	33 (20.9)	22 (21.6)	0.895
Broke into the ventricle, *n* (%)	31 (19.6)	35 (34.3)	0.008
GCS score, median (IQR)	9 (8–11)	8 (8–9)	0.000
**Prognosis**			
Rebleeding, *n* (%)	15 (9.5)	22 (21.6)	0.007
Hydrocephalus, *n* (%)	10 (6.3)	12 (11.8)	0.124
Intracranial infection, *n* (%)	13 (8.2)	8 (7.8)	0.911
Pulmonary infection, *n* (%)	41 (25.9)	45 (44.1)	0.002
Urinary tract infection, *n* (%)	11 (7.0)	3 (3.0)	0.161
**Therapeutic method**			
DTI-assisted neuroendoscopy, *n* (%)	49	20	0.042
Neuroendoscopy, *n* (%)	109	82	
Hospital stay, median (IQR), day	19 (13.8–27.3)	16 (12.0–30.5)	0.396

IQR: inter-quartile range; ICH: intracerebral hemorrhage; mRS: modified Rankin Scale; GCS: Glasgow coma scale; INR: international normalized ratio; DT: diffusion tensor imaging.

The observation indicators listed in Table 2 were analyzed using single-factor analysis, and those for which *P* < 0.1 were included in the multivariate analysis. The results showed that DTI-assisted neuroendoscopy was predictive of a good prognosis (odds ratio [OR] = 0.42, 95% confidence interval [CI] = 0.21–0.85, *P* = 0.015). The independent risk factors for an unfavorable outcome were a history of stroke (OR = 0.41, 95% CI = 0.19–0.85, *P* = 0.016), rebleeding (OR = 0.30, 95% CI = 0.14–0.68, *P* = 0.004), larger hematoma volume (OR = 1.05, 95% CI = 1.01–1.09, *P* = 0.026), and low GCS score (OR = 0.72, 95% CI = 0.55–0.94, *P* = 0.016). Taking into account the differences in baseline data between the DTI-assisted neuroendoscopy group and the standard neuroendoscopy group, the variables for which *P* < 0.05 in Table 1 were included in the multivariate analysis. The results suggested that DTI-assisted neuroendoscopy is still predictive of a favorable outcome (OR = 0.40, 95% CI = 0.20–0.82, *P* = 0.013) (shown in Table 3).

**Table 3:** Multivariate analysis of favorable outcome and unfavorable outcome

	OR (95% CI)	*P*
**Model 1**		
DTI-assisted neuroendoscopy	0.42 (0.21–0.85)	0.015
GCS score	0.72 (0.55–0.94)	0.016
History of stroke	0.41 (0.19–0.85)	0.016
Hematoma volume	1.05 (1.01–1.09)	0.026
Rebleeding	0.30 (0.14–0.68)	0.004
**Model 2**		
DTI-assisted neuroendoscopy	0.40 (0.20–0.82)	0.013
GCS score	0.72 (0.55–0.95)	0.019
History of stroke	0.40 (0.19–0.83)	0.015
Hematoma volume	1.04 (1.00–1.09)	0.039
Rebleeding	0.31 (0.14–0.70)	0.005

OR: odds ratio; CI: confidence interval; DTI: diffusion tensor imaging; GCS: Glasgow coma scale.

**Figure 2:**
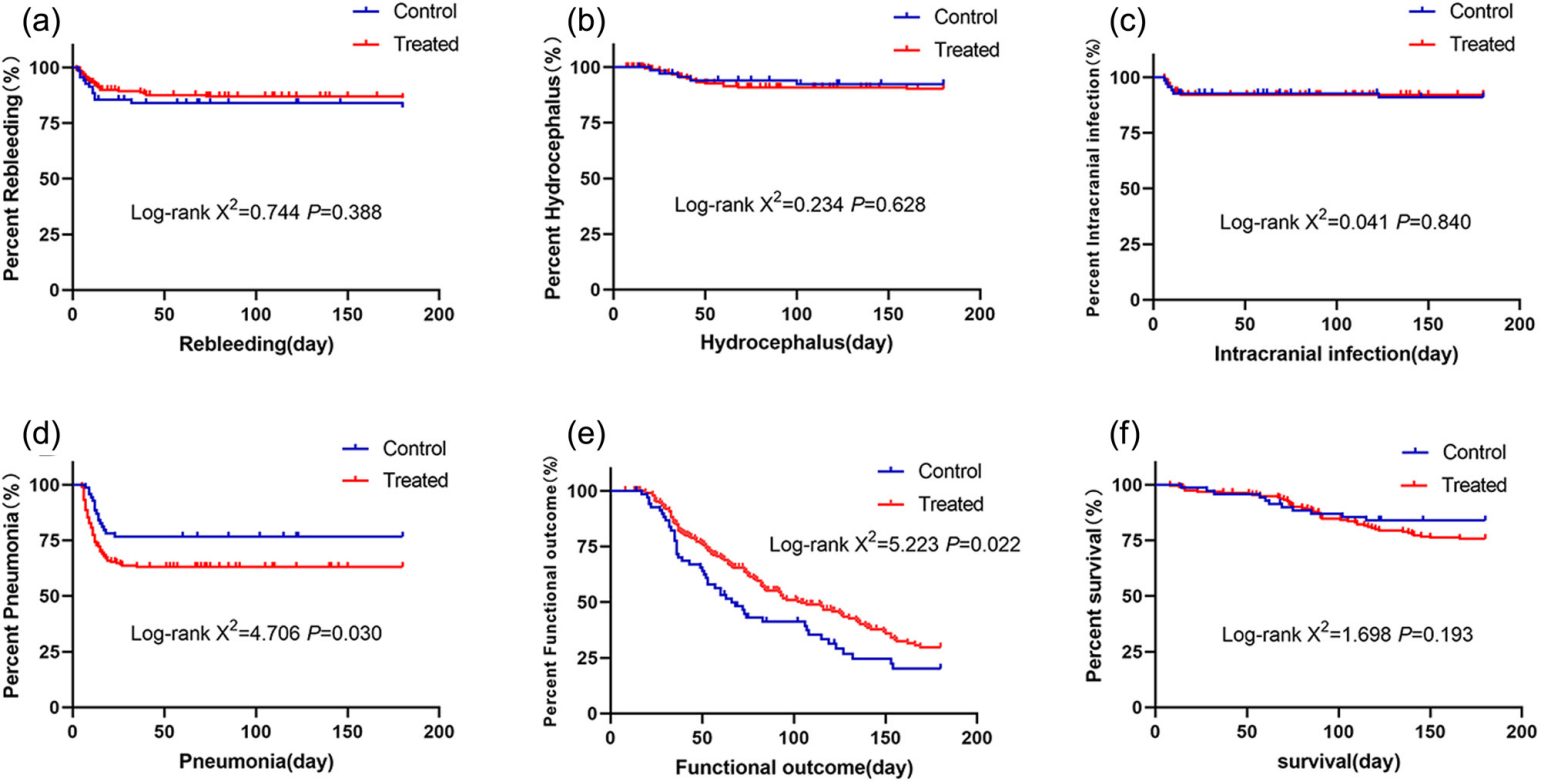
(a) Survival curve of rebleeding 6 months after operation; (b) survival curve of hydrocephalus 6 months after operation; (c) survival curve of intracranial infection 6 months after operation; (d) survival curve of pulmonary infection 6 months after operation; (e) survival curve of favorable outcome 6 months after operation; (f) survival curve at 6 months after operation.

The Kaplan–Meier survival curves showed that the rates of rebleeding, hydrocephalus, intracranial infection, and mortality did not differ significantly between the DTI-assisted neuroendoscopy group and the standard neuroendoscopy group (all *P* > 0.05). However, the median time to a favorable outcome was shorter in the DTI-assisted neuroendoscopy group (66 days, 95% CI = 48.50–83.50 days) than in the standard neuroendoscopy group (104 days, 95% CI = 75.55–132.45 days). The log-rank test showed that the pulmonary infection rate was lower (*χ* ^2^ = 4.706, *P* = 0.030) and the prognosis was better (*χ* ^2^ = 5.223, *P* = 0.022) in the DTI-assisted neuroendoscopy group (shown in Figure 2).

## 4 Discussion

There is still no standard treatment for ICH. The STICH I and II studies [3,4] showed that compared with conservative treatment, applying craniotomy to clear a hematoma does not reduce the mortality rate or improve the neurological function outcome, which might be due to craniotomy representing major trauma to the patient. Also, because craniotomy is traditionally performed using the closest approach to the hematoma, the hematoma removal and hemostasis process are not sufficiently precise, which increases the probability of iatrogenic damage to the white-matter fiber tracts [20]. However, the disease conditions of the patients included in the STICH I and II studies were quite different, with about 25% of the patients who received surgical intervention initially being treated conservatively. This crossover between groups might also have affected the results of the study [21]. The MISTIE II and III studies [8,9] were characterized by improved patient selection, and they demonstrated the effectiveness of minimally invasive surgery in the treatment of ICH. The advantages of minimally invasive surgery when removing a hematoma are not only that the intervention is less traumatic, but also that it can effectively relieve the pressure exerted by the hematoma on normal brain tissue, as well as reducing secondary damage to brain tissue due to factors such as harmful substances, cytotoxins, and free radicals in the blood [22]. Moreover, minimally invasive surgery can avoid brain damage caused by peripheral edema or even death caused by brain herniation.

The neuroendoscopy-based minimally invasive technologies applied to treat ICH are becoming increasingly mature. Some studies [23,24,25] have found that neuroendoscopic minimally invasive surgery for hematoma removal has the advantages of less trauma, shorter operation time, less intraoperative blood loss, and higher hematoma clearance rate. However, the MISTIE II and III studies [8,9] found that minimally invasive surgery did not significantly improve neurological function outcomes.

Considering that DTI can specifically reveal the spatial relationships between white-matter fiber tracts and hematomas, its use can facilitate the approaches to the white-matter fiber tracts and the longitudinal axis of the hematoma in neuroendoscopy, thereby protecting the white-matter fiber tracts and reducing secondary damage to brain tissue. Therefore, DTI-assisted neuroendoscopy may be able to reduce iatrogenic damage to the white matter during surgery and so improve the neurological function outcomes of the patients. The present findings have confirmed this conjecture. The patients in the DTI-assisted neuroendoscopy group underwent a DTI examination before surgery. Clinicians with more than 20 years of experience then designed precise surgical approaches based on the identified spatial relationships between the white-matter fiber tracts and the spatial position of the hematoma so as to avoid iatrogenic injury of the white-matter fiber tracts to the greatest extent possible. Compared with those who received standard neuroendoscopic hematoma removal, the patients in the DTI-assisted neuroendoscopy group had better prognostic mRS scores (*P* = 0.027).

While the mortality rate did not differ between our two groups of patients, which is consistent with the conclusions of other studies [8,9], the overall mortality rate was lower in the present study. This between-studies difference might have been due to critically ill patients with large bleeding volumes not being included in our study because they had contraindications for preoperative DTI examinations. When we divided patients into groups with favorable and unfavorable outcomes, the comparison results suggested that DTI-assisted neuroendoscopy is associated with a good prognosis (*P* = 0.042). In addition, no history of stroke, history of hypertension, hematoma breaking into the ventricle, postoperative rebleeding, or pulmonary infection, a small hematoma volume, and a high GCS score were also factors contributing to the good prognosis of patients with ICH, which is consistent with the conclusions of other studies. Our multivariate analysis showed that DTI-assisted neuroendoscopy is an independent predictive factor for a good prognosis (OR = 0.42, *P* = 0.015).

It is worth mentioning that in our data, the blood glucose level of patients in the DTI-assisted neuroendoscopy group was higher than that in the neuroendoscopy group, which may be an accidental error. We introduced the multivariate analysis model 2 to exclude the influence of this confounding factor. Fortunately, it didn’t have a significant impact on our results. The baseline variables in Table 1 for which *P* < 0.05 in comparisons between the DTI-assisted neuroendoscopy group and the standard neuroendoscopy group were included in the multivariate analysis, with the results showing that DTI-assisted neuroendoscopy remained predictive of a good prognosis (OR = 0.40, *P* = 0.013). The Kaplan–Meier survival curves also showed that the median time to a good prognosis in the DTI-assisted neuroendoscopy group was 66 days (95% CI = 48.50–83.50 days), which was shorter than that in the standard neuroendoscopy group (104 days, 95% CI = 75.55–132.45 days). The log-rank test further showed that the prognosis was better (*χ* ^2^ = 5.223, *P* = 0.022), and the lung infection rate was lower (*χ* ^2^ = 4.706, *P* = 0.030) in the DTI-assisted neuroendoscopy group. The reason may be that patients in the DTI-assisted neuroendoscopy group had less hospital stay and bed stay, which was beneficial to reduce the rate of pulmonary infection. At the same time, the lower rate of pulmonary infection is conducive to the rehabilitation exercise of patients earlier, which is conducive to the recovery of neurological function. It’s a virtuous circle. And they demonstrate that using neuroendoscopy supplemented with DTI to treat ICH is an effective method for reducing the disability rate of patients and improving their neurological function outcomes.

This study also had some limitations. First, our research had a retrospective design, and so there may have been deviations in the collection and selection of data. Second, our proposed methodology is not helpful for critically ill patients with large bleeding volumes (>50 mL), since such patients are often in a critical condition and hence are unsuitable for a preoperative DTI examination.

## 5 Conclusion

In summary, we believe that neuroendoscopy supplemented with DTI for the treatment of ICH (when conditions permit) is an effective way to reduce the disability rate of patients and improve their neurological function outcomes. However, further investigations are needed into how to improve the survival rate and neurological function outcomes of critically ill patients, such as those with huge bleeding, brainstem hemorrhage, and secondary cerebral hernia.
